# An Unusual Presentation of Desmoplastic Small Round Cell Tumour of the Abdomen: Morphological, Immunohistochemical, Ultrastructural, and Molecular Studies

**DOI:** 10.1155/2011/183938

**Published:** 2011-09-19

**Authors:** Preethika Angunawela, Alhossain A. Khalafallah, Karen Wolfswinkel, David Seaton, Terry Brain

**Affiliations:** ^1^Pathology Department, Launceston General Hospital, Launceston, TAS 7250, Australia; ^2^School of Human Life Sciences, University of Tasmania, Launceston, TAS 7250, Australia

## Abstract

Desmoplastic small round cell tumour (DSRCT) is an aggressive and a rare neoplasm. We report on a 34-year-old male who had abdominal discomfort with a large intraperitoneal mass. Histological examination of the tumour biopsy revealed sheets of small round cells. The cells were positive with vimentin and desmin (with occasional dot positivity) and negative for WT1 and CD 99 with immunohistochemistry. Cytogenetics showed a translocation disrupting the EWSR 1 gene on 22 q 12 consistent with DSRCT. Electron microscopic examination showed sparse cytoplasmic organelles. The patient succumbed 34 months from disease presentation after multiple chemotherapies and thereafter radiotherapy. In summary, our case exemplifies that it is crucial to combine clinical, histological, and molecular aspects in diagnosing DSRCT especially when characteristic dot positivity with desmin is weak along with deficient marking of WT1 and CD99 by immunohistochemistry. Histology was also less clear than published examples of this entity with a poor desmoplastic response. A multidisciplinary approach including early referral to specialised centres is recommended in these cases as tertiary referral centres will be required to substantiate the diagnosis.

## 1. Introduction

Desmoplastic small round cell tumour (DSRCT) is classified as a soft tissue sarcoma. It is an aggressive and rare tumour that primarily presents with an abdominal mass with only a few cases having been reported in the literature [1]. Patients' ages at diagnosis have ranged from 3 to 48 years with a mean age of 21 years [2]. 

There are no risk factors that have been identified specific to the disease. The tumour appears to arise from the primitive cells of childhood and is considered a childhood cancer. Research has indicated that there is a relationship between desmoplastic small round cell tumour, Wilm's tumour, and Ewing's sarcoma. Some DSRCT can present clinically, simulating lymphoma or other solid tumours. Therefore, careful selection of diagnostic tools should be employed to differentiate between such tumours. 

DSRCT is usually associated with a unique chromosomal translocation t(11;22) (p13:q12) resulting in an EWS/WT1 transcript [3, 4] which is diagnostic of this tumour [5]. This transcript codes for a protein that acts as a transcriptional activator that fails to suppress tumour growth.

Due to the rarity of this tumour, many general practitioners and oncologists are not familiar with the (DSRCT) entity. DSRCT in young patients can be confused with other abdominal tumours including lymphoma, rhabdomyosarcoma, neuroblastoma, primitive neuroectodermal tumour, small cell mesothelioma, Ewing's sarcoma, and Wilm's tumour. Therefore, patients with the likelihood of DSRCT should be referred to a sarcoma specialist centre where adequate expertise would be available for the complex diagnostic tests required for confirmation of the entity. 

There is no standard protocol for the treatment of the disease. However, recent literature and studies have reported that some patients respond to high-dose chemotherapy with other modalities of treatment such as tumour debulking, cytoreductive surgery, and radiation therapy [6]. Overall the prognosis for DSRCT is poor with less than 20% surviving beyond two to three years [7].

## 2. Case Study

We report on a 34-year-old male who presented in January 2008 to the Launceston General Hospital, Tasmania with 6-month history of abdominal discomfort and pain on lying on his stomach when surfing. There was no other significant past medical history other than tonsillectomy. He was a social smoker and occasionally consumed alcohol. He had a major weight loss from July 2007 until January 2008. He did not have night sweats or any change in his appetite. On examination, he was found to have a painful swelling in the upper left quadrant of his abdomen. The hard mass was about 15 cm in diameter, extending down towards the umbilicus.

His full blood count was normal. The liver and renal function tests were normal. He was negative for hepatitis B and C viruses as well as for HIV. His serum CA 125 level was slightly high at 65 U/mL (Normal <35) with other tumour markers being within normal range except for serum lactate dehydrogenase (LDH), which showed a mild increase. He had evidence of past infection with Epstein Barr virus. The cerebrospinal fluid examination did not reveal any abnormal lymphoid or malignant cells. On computer tomography (CT) scan, he was found to have a large cystic intraperitoneal mass with a suspicion of involvement of abdominal lymph nodes. The soft tissue density was present in the area of the pancreas, porta hepatis, para-aortic region and with extension to the thorax where rounded densities over the pericardial region were noted.

Two CT-guided biopsies were performed in January and in early February 2008. The first biopsy revealed scanty material and showed only soft connective tissue. The second biopsy had a small piece of fibrous tissue infiltrated by sheets of small cells resembling lymphoid cells. Immunohistochemistry with lymphoma panel markers were negative, and the tissue sample was not sufficient for further studies. Subsequently, in February 2008, he underwent open abdominal surgery, and a large mass in relation to the pancreas was excised. The tumour was extending into the lesser sac and into the right subphrenic space. Omentum was also involved. Complete resection was not possible due to its widespread tissue involvement. The resected specimen had three lobulated greyish masses measuring 4 × 3 × 2 cms. The cut surface was soft, greyish, and uniform. Solid and semi-cystic components were present. Frozen sections showed a small blue round cell tumour. The flow cytometry suggested a nonhaematological malignancy.

**Figure 1:**
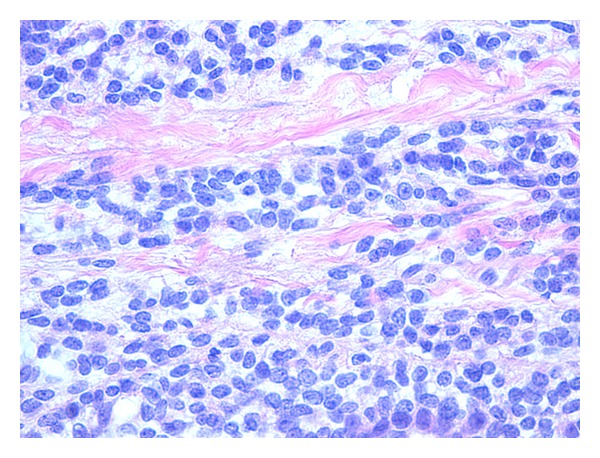
Tumour tissue. There are sheets of small round cells separated by fibrous tissue (H&E).

Histologically, the tumour was composed of large sheets of small round cells forming lobulated nodules (Figure 1). The nuclei were small, hyperchromatic with indistinct nucleoli and irregular nuclear membranes. There were also some nuclear grooves and a few oval nuclei. There was only minor variation in nuclear size. Mitotic figures were sparse. No obvious necrosis was present. There were numerous ectatic type vessels within the tumour. At the periphery of the tumour nodules there was a minimal desmoplastic stroma between sheets of tumour cells. The microscopic appearances suggested a small blue cell tumour with the differential including DSRCT.

**Figure 2:**
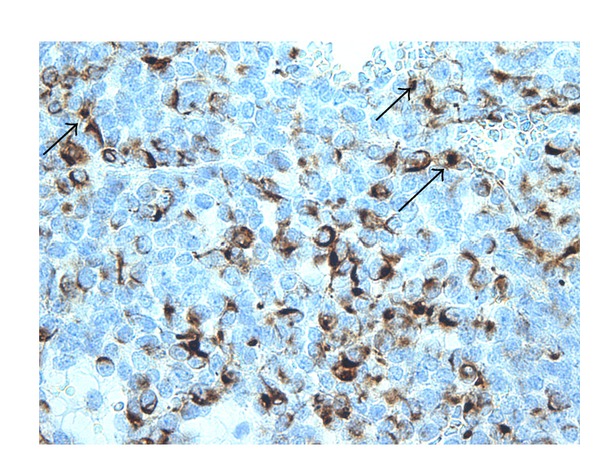
Immunohistochemistry with desmin: perinuclear dot-like staining (arrow).

Immunohistochemical stains showed a patchy perinuclear staining pattern with desmin, but characteristic dot-positivity was not prominent (Figure 2). There was focal mild positive staining with neuron-specific enolase (NSE) and moderate staining with AE1/AE3 along with some patchy dot positivity with CAM 5.2. There was negative staining with smooth muscle actin, calretinin, CD 45, chromogranin A, TTF1, synaptophysin, and S100. EMA showed only less than 5% of cells staining positive. The tumour cells were negative with WT1 and CD 99. Although there was initial clinical suspicion of lymphoma, CD 45 and other lymphoid markers were negative. Strong positivity with vimentin and patchy perinuclear region staining with desmin suggested that this lesion is likely to be a DSRCT.

Cytogenetic studies showed disruptions in the Ewing's sarcoma gene (*22p 11 q), Wilm's tumour gene by FISH probe, and RT-PCR studies were repeated at the Sullivan Nicolaides Pathology, Queensland and showed a positive result with the Ewing's sarcoma dual-colour break-apart probe. This finding is consistent with DSRCT. 

**Figure 3:**
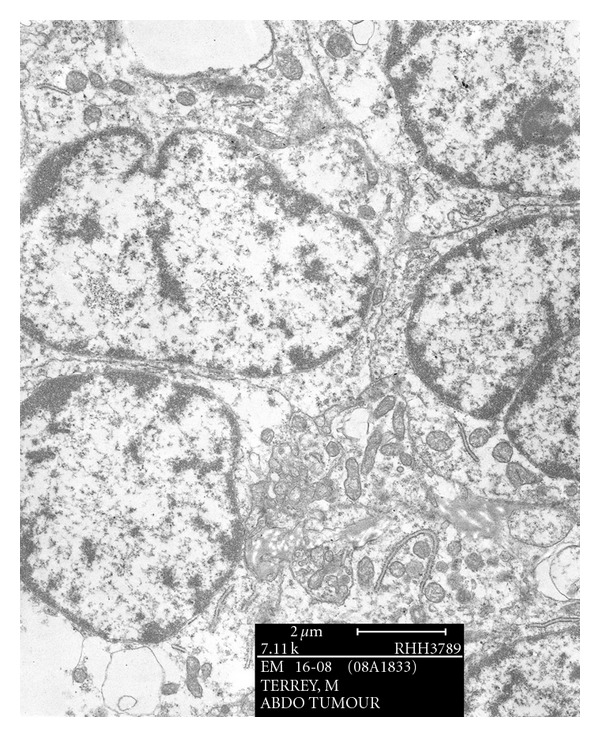
Electron microscopy showing sparse cytoplasmic organelles with short strips of rough endoplasmic reticulum and mitochondria.

Electron microscopic examination showed a population of primitive cells. The cytoplasmic organelles were sparse with short strips of rough endoplasmic reticulum and occasional mitochondria. The characteristic whorls of intermediate filaments usually present in DSRCT were not identified, and there were no dense core cytoplasmic granules (Figure 3). The ultrastructural features are consistent with an undifferentiated small cell tumour. 

Due to the negative immunohistochemical markers for WT1 and CD99 with positive findings for desmin and vimentin it was still thought compatible with a DSRCT even though histologically desmoplasia was minimal. The histology and immunohistochemistry were forwarded for review to two specialist centres because of ambiguity with the proposed diagnosis.

## 3. Discussion

The most acceptable consensus is that this case represents a small round blue cell tumour without evidence of significant desmoplasia but should nonetheless be classified as a DSRCT. Moreover, the cells were negatively stained with both WT1 and CD 99, which is against DSRCT. However, Lae et al. [8] reported that WT1-positive tumours were found in 91% and CD99-positive tumours only in 23%. The unusual finding here in our patient was that both WT 1 and CD 99 were negative and that desmoplasia was minimal.

In a histopathological study [9] of 39 cases of DSRCT most cases had shown the characteristic pattern of small blue cells embedded in a dense fibrous stroma. About one-third of tumours exhibited a wide range of morphological features such as a predominant component of spindle cells, presence of Homer-Wright-like, rosettes and an insular pattern. The recognition of these uncommon morphological variants of DSRCT is of paramount importance to avoid misdiagnosis as these tumours can be confused with other neoplastic lesions.

In summary, we present a difficult case of a 34-year-old man with an abdominal tumour simulating lymphoma. The morphological pattern and immunohistochemistry favour a diagnosis of DSRCT, but with less desmoplasia than is usual. Cytogenetics performed on fresh tissue with FISH showed a translocation disrupting the EWS gene on 22p 11q, with an EWS-WT1 gene fusion. The repeat FISH probe and RT-PCR studies performed at the Sullivan Nicolaides Pathology showed a positive result with Ewing's sarcoma dual-colour break-apart probe. This finding is consistent with DSRCT. Electron microscopic studies were consistent with an undifferentiated small cell tumour as dense core granules, characteristic of DSRCT, were not present. The patient was referred to the Peter MacCallum Cancer Centre in Melbourne for further management. He was treated initially according to the VAC protocol [7], thereafter with multiple chemotherapies and radiotherapy because of widespread disease to the abdomen, liver, lung, and bones. He died 28 months following the initial treatment (34 months after presentation).

For diagnosis of DSRCT a combination of clinical, histological, and molecular aspects is required especially when usual markers for the entity WT1 and CD99 are negative. As desmoplasia is minimal and characteristic desmin dot positive is scanty in our case we suggest all the above findings indicate a variant of DSRCT. Finally a multidisciplinary approach including early referral to a specialised centre is recommended in such complex cases.
